# Determining late predictors of outcome for acetaminophen- induced acute liver failure using classification and regression tree modeling analysis

**DOI:** 10.1186/cc13389

**Published:** 2014-03-17

**Authors:** C Karvellas, J Speiser, W Lee

**Affiliations:** 1 University of Alberta, Edmonton, Canada; 2 Medical University of South Carolina, Charlston, SC, USA; 3 University of Texas Southwestern, Dallas, TX, USA

## Introduction

Liver transplantation (LT) in acetaminophen-induced acute liver failure (APAP-ALF) patients often presents significant challenges. The King's College Criteria (KCC) have been validated on admission but not in later phases of illness. The aim was to improve determinations of prognosis on and after admission in APAP- ALF patients using the classification and regression tree (CART) methodology to construct optimal binary splits on independent variables to predict outcome.

## Methods

CART models were applied to US ALFSG registry data for prediction of 21-day spontaneous survival on admission and late stage (days 3 to 7). Analyses were carried out using R software (package rpart) for all (*n *= 803) APAP-ALF patients enrolled between January 1998 and September 2013 with complete outcome data. Training data were used to build CART trees and test data were used to evaluate prediction accuracy (AC), sensitivity (Sn) and specificity (Sp).

## Results

Of the 803 APAP-ALF patients, the median age was 37 (29 to 47) years and 76% were female. A total of 238 (30%) patients were listed for and 87 (11%) received LT. A total of 531 (66%) patients recovered without LT at 21 days. Using standard logistic regression methods for all patients with complete data (*n *= 679), KCC (INR, creatinine, coma grade 3/4, pH) yielded an AC of 69% (Sn 90%, Sp 27%) at admission. For late-stage data (*n *= 341), KCC provided similar AC (70%) and Sn (97%), but suffered poor Sp (15%). CART analysis using the KCC variables on admission offered predictive AC (66%) and Sn (65%), with increased Sp (67%). Using day 3 to 7 data, the KCC-CART model had increased AC (82%) and Sn (86%), with improved Sp (46%) compared with logistic KCC. New CART models were developed with 18 variables based on previous literature. A new CART model on admission (MELD, lactate and MV: test AC 72%, Sn 71%, Sp 77%) performed better than KCC-CART. For days 3 to 7, a CART model (MELD, lactate, coma grade) offered superior prediction (test AC 86%, Sn 91%, Sp 46%) compared with days 3 to 7 KCC-CART.

## Conclusion

CART analysis increased predictive performance compared with traditional KCC. KCC-CART trees have higher Sp and similar predictive AC compared with traditional KCC, with newer CART trees providing marginal improvement over KCC-CART models.

